# Low‐Power Negative‐Differential‐Resistance Device for Sensing the Selective Protein via Supporter Molecule Engineering

**DOI:** 10.1002/advs.202204779

**Published:** 2022-11-14

**Authors:** Ghulam Dastgeer, Sobia Nisar, Zafar Muhammad Shahzad, Aamir Rasheed, Deok‐kee Kim, Syed Hassan Abbas Jaffery, Liang Wang, Muhammad Usman, Jonghwa Eom

**Affiliations:** ^1^ Department of Physics and Astronomy Sejong University Seoul 05006 Korea; ^2^ Department of Electrical Engineering Sejong University Seoul 05006 Korea; ^3^ Department of Chemical & Polymer Engineering University of Engineering and Technology Lahore, Faisalabad Campus 38000 Pakistan; ^4^ SKKU Advanced Institute of Nanotechnology (SAINT) Sungkyunkwan University Suwon 16419 Korea; ^5^ Department of Physics and Interdisciplinary Course of Physics and Chemistry Sungkyunkwan University Suwon Gyeonggi‐do 16419 Korea; ^6^ HMC (Hybrid Materials Center) Department of Nanotechnology and Advanced Materials Engineering and Graphene Research Institute Sejong University Seoul 05006 Korea; ^7^ Department of Bioinformatics School of Medical Informatics and Engineering Xuzhou Medical University Xuzhou 221006 China

**Keywords:** broken bandgap, negative differential resistance, selective protein detection, van der Waals heterostructure

## Abstract

Van der Waals (vdW) heterostructures composed of atomically thin two‐dimensional (2D) materials have more potential than conventional metal‐oxide semiconductors because of their tunable bandgaps, and sensitivities. The remarkable features of these amazing vdW heterostructures are leading to multi‐functional logic devices, atomically thin photodetectors, and negative differential resistance (NDR) Esaki diodes. Here, an atomically thin vdW stacking composed of p‐type black arsenic (b‐As) and n‐type tin disulfide (n‐SnS_2_) to build a type‐III (broken gap) heterojunction is introduced, leading to a negative differential resistance device. Charge transport through the NDR device is investigated under electrostatic gating to achieve a high peak‐to‐valley current ratio (PVCR), which improved from 2.8 to 4.6 when the temperature is lowered from 300 to 100 K. At various applied‐biasing voltages, all conceivable tunneling mechanisms that regulate charge transport are elucidated. Furthermore, the real‐time response of the NDR device is investigated at various streptavidin concentrations down to 1 pm, operating at a low biasing voltage. Such applications of NDR devices may lead to the development of cutting‐edge electrical devices operating at low power that may be employed as biosensors to detect a variety of target DNA (e.g., ct‐DNA) and protein (e.g., the spike protein associated with COVID‐19).

## Graphical abstract

In this article, the authors report an atomically thin vdW stacking of “b‐AS/SnS_2_,” to build a type‐III (broken gap) heterojunction, leading to a negative differential resistance (NDR) device. The hexagonal surface of SnS_2_ offers an ideal plate‐form of *π*–*π* stacking for supporter molecules composed of a “pyrene ring” loaded with “lysine‐biotin” to detect the “streptavidin” biomolecules as analytes.

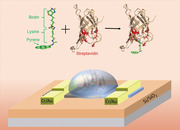

## 1 Introduction

Van der Waals (vdW) heterostructures comprising two‐dimensional (2D) transition‐metal dichalcogenide (TMDC) materials have been utilized to manufacture multi‐functional electronic devices such as memory devices, photodetectors, photovoltaics, and light‐emitting diodes. ^[^ ^1^, ^2^, ^3^, ^4^, ^5^ ^]^ The charge transportation through these vdW heterostructures depends on the bandgaps of the materials, their interfaces, and the metal contacts. Bandgap engineering of 2D vdW heterostructures offers a wide range of smart devices, such as p–n junction diodes, logic devices, and negative differential resistance (NDR) devices, which are used for various potential applications.^[^ ^6^, ^7^, ^8^, ^9^, ^10^, ^11^, ^12^ ^]^ Type‐I (straddling gap) and type‐II (staggered gap) p–n junctions are usually used for rectification, logic switching, and photovoltaic applications. ^[^ ^13^, ^14^, ^15^, ^16^, ^17^, ^18^ ^]^ Typically, vdW heterostructures with type‐III (broken gap) band alignment are employed to build NDR devices used for multivalued logic operations. NDR devices are noteworthy because they have numerous threshold voltages and a higher current value than conventional p–n junction diodes.^[^ ^19^ ^]^ These devices are famous as Esaki diodes, which are composed of heavily doped semiconductor materials holding a large current density, prompt triggering speed, and low operating power.^[^ ^20^, ^21^ ^]^

Just after the discovery of gapless graphene, a large number of atomically thin 2D layered materials have been discovered, with bandgap values varying from 0.3 to 2 eV, which allows electrostatic control of a large number of holes or electrons.^[^ ^5^, ^7^, ^22^, ^23^, ^24^, ^25^, ^26^, ^27^, ^28^ ^]^ A suitable stacking selection of these 2D TMDC semiconductors may offer sharp and atomically thin vdW heterostructures, with minimal lattice mismatch, which could be used to build multi‐functional electronic and optoelectronic devices. Recently, a type‐II dual‐gated NDR device composed of a MoS_2_/WSe_2_ vdW heterostructure was reported. The complex fabrication process and dual‐gate control limited its performance, although it exhibited NRD behavior below a temperature of 175 K. ^[^ ^29^ ^]^ In another study, a BP/SnSe_2_ heterostructure displayed NDR behavior, but the peak‐to‐valley current ratio (PVCR) was lower than 2. Both materials employed to construct an NDR device were highly susceptible to oxidation in the presence of air, resulting in a small PVCR value.^[^ ^9^ ^]^ The NDR devices reported in these two former studies have a limited role to deploy in the electronics industry. Moreover, the potential implementation of NDRs in the field of biomolecule detection has not been explored yet, even though NDR devices with vdW heterostructures exhibit superior performance with a fast photo‐response.

In this study, we introduced an atomically thin vdW stacking of the p‐type black arsenic (b‐As) over the n‐type tin disulfide (n‐SnS_2_) to build a type‐III (broken gap) heterojunction that demonstrated Esaki‐diode (NDR) behavior at room temperature. Charge transport through the vdW heterostructure was investigated under electrostatic gating control to achieve a high PVCR, which improved from 2.8 to 4.6 when the temperature was lowered from 300 to 100 K. At various applied‐bias voltages, all conceivable tunneling mechanisms that regulate the charge transport were extensively elucidated. Furthermore, an NDR device was deployed as a biosensor for the first time to detect the selective protein as an analyte in a short time (12.5 s), anticipating its potential application. The real‐time response of the NDR device was investigated at several target protein concentrations ranging from 100 to 1 pm. Even at low biasing voltages, the NDR device was functionalized to achieve the precise and prompt detection of streptavidin up to 1 pm. Such applications of vdW NDR devices may lead to the development of cutting‐edge electrical devices that may be employed as biosensors to detect a variety of target DNA and protein molecules, such spike protein associated with COVID‐19.

## 2 Results and Discussion

**Figure 1:**
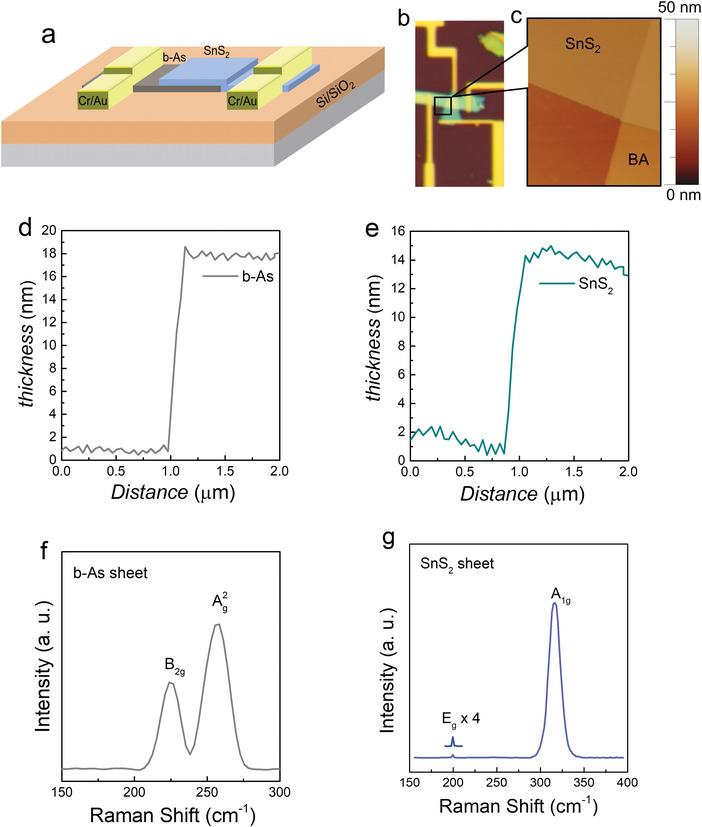
Schematics and basic characterization of the NDR device. a) A schematic illustration of the NDR device composed of p‐type b‐As (dark grey color) and n‐type SnS_2_ (sky blue color) sheets to form a vdW heterostructure. b) An optical image with a scale bar of 10 µm and c) atomic force microscope (AFM) image of the black marked region (5 × 5 µm^2^) show the smooth and sharp interface. d) The thickness of the bottom b‐As sheet is ≈17 nm and e) the thickness of the SnS_2_ sheet is ≈14 nm, which was confirmed by the height profile graph extracted from the AFM image. f) Raman spectrum obtained for the multi‐layer b‐As and g) Raman spectrum obtained for the multi‐layer SnS_2_. The calculated full width at half maximum (FWHM) of the main resonance peak (*A*_2g_) for b‐As and (*A*_1g_) for SnS_2_ appears to be 17 and 9.85 cm^−1^, respectively. The peaks were fitted by Gaussian functions.

To fabricate the vdW heterostructure of b‐As/SnS_2_, high‐quality crystals of both materials were obtained from the manufacturer (HQ Graphene) and exfoliated using a piece of transparent Scotch tape. A few‐layer‐thick sheet of b‐As (≈14 nm) was mechanically exfoliated and transferred over the p‐doped SiO_2_/Si substrate first, followed by a few‐layer‐thick sheet of SnS_2_ (≈18 nm) exfoliated and transferred over the b‐As. Cr/Au metal electrodes were deposited via thermal deposition for big electrodes and microelectrodes following the conventional photolithography and electron lithography techniques, respectively (see details in the device fabrication section). The schematic and optical images of the final device are represented in **Figure** **1**a,b, respectively. The surfaces of the b‐As and SnS_2_ sheets and their thicknesses were analyzed via atomic force microscopy (AFM), as shown in Figure 1c. The thickness of b‐As was 14 nm, while that of SnS_2_ was 18 nm, as confirmed by the height‐profile graphs shown in Figure 1d,e. Raman analysis was performed using a green laser with a wavelength of 532 nm to assess the quality of the b‐As and SnS_2_ sheets. The Raman spectra obtained from the b‐As sheet showed Raman peaks *B*_2g_ and *A*_2g_ at 225 and 257 cm^−1^, respectively.^[^ ^30^, ^31^ ^]^ Here, *B*_2g_ represents the out‐of‐plane vibrations of the material and *A*_2g_ represents the in‐plane vibrations.

Furthermore, the Raman spectra recorded from the SnS_2_ sheet exhibits the peaks *E*_g_ and *A*_1g_ at 206 and 315 cm^−1^, respectively, indicating the pristine nature of the material (as demonstrated in Figure 1f). These peaks represent two Raman‐active phonon modes of 2H‐phase SnS_2_.^[^ ^32^ ^]^ The presence of a single peak (*E*_g_) in the 190–225 cm^−1^ range confirms the fine crystal quality^[^ ^33^ ^]^ of SnS_2_, whereas the sharp resonance peak (*A*_1g_) at 315 cm^−1^ represents multi‐layer SnS_2_. Moreover, the pristine nature of the SnS_2_ material was also verified by the excellent intensity ratio (*A*_1g_ */ E*_g_ > 100).

**Figure 2:**
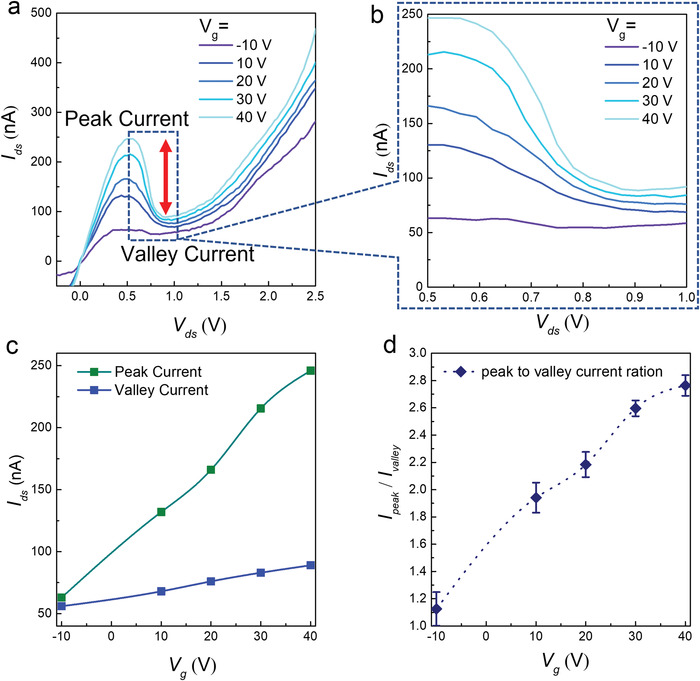
Electrical characterization of the NDR device as a function of gate voltages. a) Output curves (*I*_ds_ *−V*_ds_) obtained at various gate voltages varying from *V*_g_ = –10 to +40 V, illustrate an excellent NDR trend at room temperature. b) The *I*_ds_−*V*_ds_ curves in the small range of applied bias voltage show a maximum value of the peak current at *V*_g_ = 40 V. c) The peak current (in cyan color) and valley current (in blue color) are plotted as a function of gate voltage. d) The peak‐to‐valley current ratio (PVCR) plotted at various gate voltages. The maximum PVCR value was obtained at *V*_g_ = 40 V. The data were taken from 3 similar devices.

The electrical measurements for each material were performed individually to investigate their intrinsic nature. The transfer curves of SnS_2_ and b‐As are shown in Figures S1a,b (Supporting Information), revealing their n‐ and p‐type nature, respectively. The hole carrier density in b‐As increased at a negative gate voltage, whereas the electron carrier density exhibited the reverse trend. The non‐ohmic output curves for SnS_2_ and b‐As were measured at zero gate voltage, as presented in Figures S1c,d (Supporting Information). To study the electric transport through the vdW heterostructure of b‐As/SnS_2_, the source–drain current (*I*_ds_) was measured as a function of the bias voltage (*V*_ds_) at various gate voltages. **Figure** **2**a demonstrates the evident NDR behavior on a linear scale in the gate‐dependent *I*_ds_−*V*_ds_ curves at room temperature. At a higher positive gate voltage, the Fermi level of the n‐type SnS_2_ shifted upward owing to the growing accumulation of electrons, whereas the Fermi level of the p‐type b‐As shifted downward. This higher electrostatic gating aligns the Fermi levels of n‐type SnS_2_ and p‐type b‐As closer to each other, and band‐to‐band tunneling (BTBT) occurs, which is responsible for the excellent NDR peak. ^[^ ^19^, ^20^ ^]^ The peak current decreased as the gate voltage changed to a more negative region and increased as the gate voltage increased in the positive regime. When the gate voltage increased from –10 to +40 V, a considerable variation in the peak current was observed, as shown in Figure 2b. The b‐As/SnS_2_ NDR device exhibited a small observable peak at *V*_g_ = –10 V, which saturated above the gate voltage of +40 V. This gate‐dependent change in the peak current is attributed to the Fermi‐level shift in b‐As, which moves downward because of charge accumulation at the interface. There is no significant gate‐dependent shift of the Fermi level in SnS_2_ because of the strong screening effect, as it is stacked over b‐As. ^[^ ^19^, ^20^, ^21^ ^]^ The gate‐dependent peak current, valley current, and their ratios are presented in Figure 2c,d. There is a large variation in the peak current as a function of the gate voltage, but only a slight variation in the valley current was observed when the gate voltage was changed from –10 to + 40 V. The significant variation in peak current as a function of gate voltage is because of the BTBT which is sufficiently modulated via gate voltage. In contrast, as the biasing voltage is increased further, the forward current decreases gradually because there is no more BTBT tunneling which is ascribed to the valley current. In this region gate voltage does not affect the valley current more significantly. Figure 2d shows the PVCR as a function of the gate voltage at room temperature. A significantly high gate‐dependent value of PVCR = 2.8 was obtained at *V*_g_ = +40 V for b‐As/SnS_2_ and the heterostructure, which is sufficiently larger than the previously reported NDR devices.^[^ ^9^, ^20^, ^29^ ^]^ The gate‐dependent Fermi‐level shift in b‐As leads to the formation of a potential well at the interface of b‐As/SnS_2_ which can trap electrons or limit their transportation. These limitations to charge transportation are considered to be responsible for the change in the PVCR.

**Figure 3:**
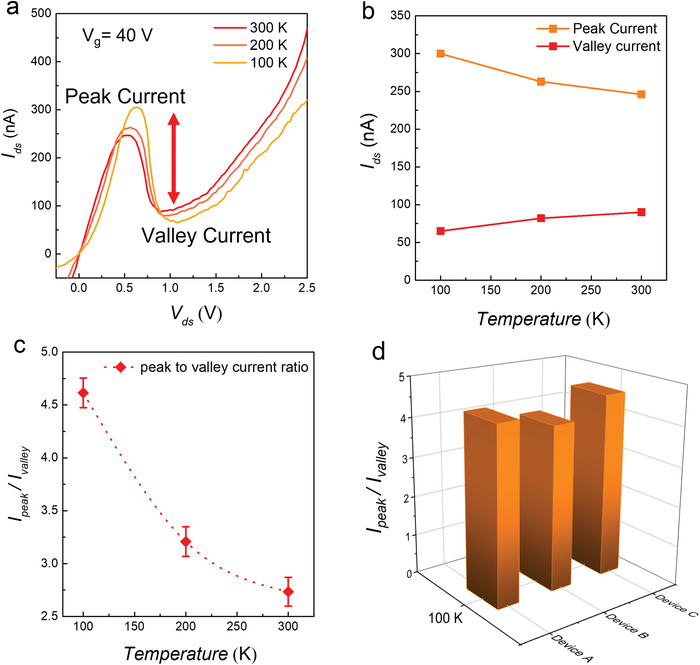
Electrical transport across the NDR device as a function of temperature. a) At a fixed gate voltage of *V*_g_ = 40 V, the *I*_ds_−*V*_ds_ curves for a b‐As/SnS_2_ NDR device were plotted at various temperatures ranging from 100 to 300 K. When the temperature decreased from 300 to 100 K, the peak current increased. b) The peak current (in orange color) and valley current (in red color) plotted as a function of temperature, at *V*_g_ = 40 V. c) The ratio of peak current over valley current (PVCR) plotted at 100, 150, 200, 250, and 300 K. At 100 K, PVCR = 4.6 reaches its highest value. The PVCR value varies as a function of temperature, as seen by the red dotted line. d) The PVCR value of three devices plotted in a bar graph at 100 K, showing consistent characteristics of the NDR devices composed of b‐As/SnS_2_ vdW heterostructures. The data were triplicated using three different devices (*n* = 3) and it was estimated that *p* ≤ 0.0005 and MSSD ≤ 0.00006 for all sets of measurements.

Furthermore, electric transport through the b‐As/SnS_2_ NDR device was investigated at different temperatures ranging from 300 to 100 K. The *I*_ds_ values were recorded on a linear scale as a function of *V*_ds_ at 300, 200, and 100 K at a fixed gate voltage of *V*_g_ = 40 V. Independent of temperature, it was observed that all the devices exhibited an excellent NDR trend. **Figure** **3**a shows that the forward current decreased as the temperature decreased from 300 to 100 K; however, the peak current increased as the temperature decreased. Regarding conventional devices, the electric current, which increases linearly with temperature, is attributable to Fowler–Nordheim (FN) tunneling and thermionic emission (TE), whereas BTBT displays the reverse tendency. ^[^ ^20^, ^34^, ^35^ ^]^ FN tunneling and TE were confirmed via a plot of ln(*I*_ds_ */V*_ds_ *^2^*) versus *1/V*_ds_ curves measured at various temperatures, as shown in Figure S2a,b (Supporting Information). When the temperature dropped to 100 K, the contribution of TE was suppressed, which caused a decline in temperature. As the BTBT mechanism is primarily responsible for the peak current, this current increases as the temperature decreases. The valley current showed a negligible surge with increasing temperature, but the peak current showed a reverse trend, as shown in Figure 3b. The Fermi–Dirac distribution curve approaches to a step function at the Fermi level when the temperature falls from 300 to 100 K, increasing the probability that electrons will occupy energy levels closer to it. This Fermi‐level shifting ultimately improves the BTBT current since the filled states are increased below the SnS_2_ Fermi level while having more empty states in b‐As. This improved BTBT at lower temperature causes an increase in peak current.^[^ ^9^, ^19^, ^20^ ^]^

Secondly, the contact resistance also plays an important role to increase the peak current at low temperature. At lower temperature, the metal/2D semiconductor resistance increases to form a higher Schottky junction which decreases the reverse and well as forward current but improves the peak current in the NDR devices.^[^ ^9^, ^20^ ^]^ To estimate the PVCR as a function of temperature, we investigated the transfer curves at a gate voltage of +40 V. As the temperature decreased from 300 to 100 K, the PVCR value increased from 2.8 to 4.6, as illustrated in Figure 3c. In Figure 3d, a bar graph depicts the PVCR values obtained from three different NDR devices at 100 K. All three NDR devices were nominally composed of the same thickness, exhibiting similarly high PVCR values.

**Figure 4:**
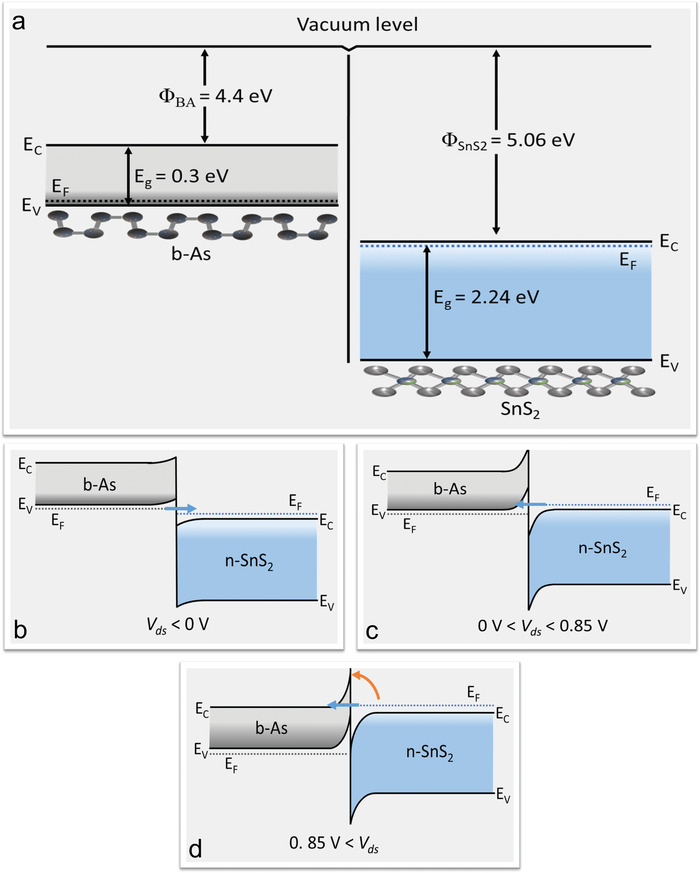
Band alignment of p‐type b‐As/n‐type SnS_2_ vdW heterostructure. a) The electron affinity and band gap values for b‐As (SnS_2_), obtained from the vacuum level before constructing a vdW heterostructure were 4.4 eV (5.06 eV) and 0.3 eV (2.24 eV), respectively. The Fermi‐level (black dotted line) of the p‐type b‐As exists near its valence band (*E*_v_) while the Fermi‐level (blue dotted line) of n‐type SnS_2_ lies near its conduction band (*E*_c_). The difference between the energy bands indicates a broken band gap (type III) vdW heterostructure. b) The band alignment at *V*_ds_ < 0 V shows the band tunneling of carriers from the conduction band of SnS_2_ to the valence band of b‐As, c) while at 0 V < *V*_ds_ < 0.85 V the carriers directly tunnel from SnS_2_ to b‐As because of overlapping. d) Under a 0.85 V < *V*_ds_ condition, TE (orange arrow line) and FN (blue arrow line) tunneling (through the triangular region) are the major mechanisms for charge transport through the NDR device.

The physical mechanism of charge transport through the b‐As/SnS_2_ vdW heterostructure is explained by the energy‐band diagram. The energy gap (*E*_g_) and electron affinity (*Φ*) of few‐layer thick pristine b‐As were measured at 0.3 and 4.4 eV, respectively, from the vacuum level,^[^ ^30^, ^36^, ^37^ ^]^ whereas the energy gap and electron affinity of few‐layer thick pristine SnS_2_ were recorded at 2.24 and 5.06 eV, respectively.^[^ ^38^, ^39^, ^40^, ^41^ ^]^ Before the formation of the vdW heterostructure, the Fermi levels of b‐As and SnS_2_ lie near the valence and conduction bands, respectively. **Figure** **4**a illustrates the formation of the broken bandgap (type III) vdW heterostructure at *V*_ds_ = 0 V, because the valence band of b‐As is located above the conduction band of SnS_2_. This broken gap creates an adequate working function (0.36 eV between SnS_2_ and b‐As, as well as electron–hole accumulation at their interface). This carrier accumulation near the interface of b‐As and SnS_2_ results a highly doped n+/p+ heterojunction without any extra chemical or electrostatic doping.

After the formation of a vdW heterostructure composed of p‐type b‐As and n‐type SnS_2_, the Fermi level aligns below the *E*_v_ of p‐type b‐As and above the *E*_c_ of n‐type SnS_2_ because of the broken gap. Charge transport through the vdW heterostructure is categorized into three regions: i) *V*_ds_ < 0 V, ii) 0 V < *V*_ds_ *< V*_peak_, and iii) *V_ds_* *>* *V*_peak_. When *V*_ds_ < 0 V, as shown in Figure 4b, the NDR device becomes reverse‐biased and the Fermi level of SnS_2_ shifts close to the Fermi level of b‐As. Under this biasing condition (*V*_ds_ < 0 V), electrons tunnel to the empty conduction band of SnS_2_ from the filled valence band of b‐As to recombine with holes, which is known as forward BTBT. ^[^ ^9^, ^19^ ^]^ The electric transport through the b‐As/SnS_2_ van der Waals heterostructure occurs because of their Fermi‐level difference Δ*E* *=* *qV*. When a small bias voltage (0 V < *V*_ds_ *< V*_peak_) is applied, the electrons from the conduction band of SnS_2_ begin to tunnel into the vacant states of the b‐As valence band because of the reverse BTBT, as shown in Figure 4c. This tunneling of electrons causes a gradual increase in the current until all empty states are filled, and the gap between the Fermi levels of both materials increase because of band bending.^[^ ^19^, ^20^ ^]^ As the biasing voltage is increased further (*V*_peak_ *<* *V*_ds_), the forward current decreases gradually until it reaches to a minimum value (valley current) because there is no more BTBT tunneling, and the NDR device exhibits an excellent NDR trend.^[^ ^9^, ^19^, ^20^ ^]^ Finally, when the biasing voltage is further increased, a threshold in the forwarding current is observed. This rise in current is attributed to TE, which is illustrated by an orange arrow line, and FN tunneling (represented by the blue arrow line) through the triangular region of the b‐As^[^ ^20^, ^34^ ^]^ as shown in Figure 4d. TE and FN tunneling were confirmed via the *I*_ds_−*V*_ds_ curve at various temperatures and their ln(*I/V* ^2^) versus 1*/V* plot, as shown in Figure S2 (Supporting Information). At higher bias voltages, the charge carriers diffused through SnS_2_ to b‐As directly because the depletion layer in the b‐As/SnS_2_ heterojunction was significantly reduced.^[^ ^21^ ^]^

**Figure 5:**
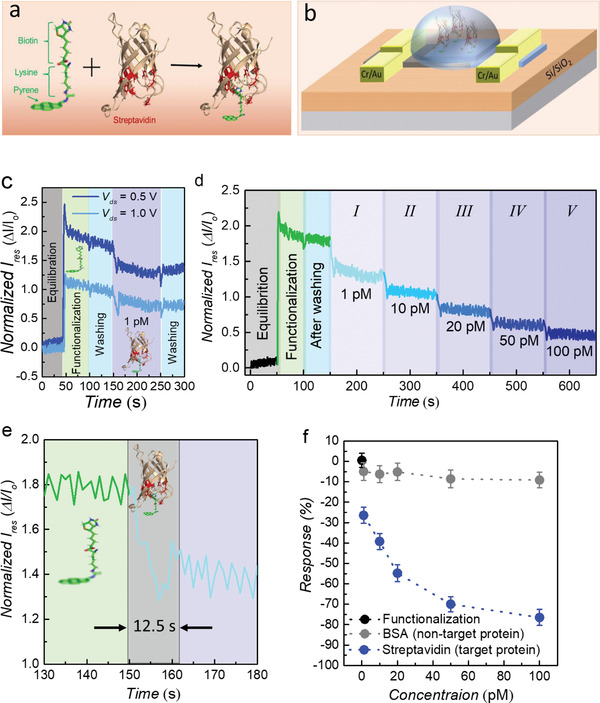
Configuration and detection mechanism of protein via NDR device. a) Schematic depiction of the supporter molecule (pyrene ring loaded with lysine and biotin) and target protein (streptavidin). The combined structure illustrates the coupling between the supporter molecule and streptavidin. Streptavidin is captured by biotin due to its inherent high‐affinity value (Ka = 2.5 × 10^13^ m ^−1^). b) NDR device schematic showing the measurement configuration of the NDR device as a biosensor. After device functionalization, a small drop of the solution containing streptavidin is drop‐casted over the NDR device, which is captured by the supporter construct. c) The real‐time measurements at *V*_ds_ = 0.5 V (in blue color) and *V*_ds_ = 1 V (in cyan color) are presented with a normalized current to check the NDR device's response against streptavidin. d) Real‐time response of the NDR device at a fixed *V*_ds_ = 0.5 V. The concentration of the streptavidin was varied down to 1 pm. The NDR device response after functionalization with supporter molecules (PLB) is shown in green, while the real‐time response to detect streptavidin concentrations ranging from 1 to 100 pm is shown in cyan and navy‐blue colors, respectively. e) The ultimate real‐time response of the NDR device for 1 pm streptavidin recorded for up to 12.5 s. f) The percentage response of the NDR device recorded for various concentrations of the target protein (streptavidin) and non‐targeted biomolecule (BSA). The percentage response is equilibrated as a decrease in response current of the NDR device. For all measurements, the variance was significant (*p* ≤ 0.05) with *σ* ≤ 0.2, IQR ≤ 0.4, and MSSD ≤ 0.02.

Finally, the NDR devices were successfully utilized to detect the selective proteins at the sub‐molar level. A similar NDR device strategy can be used to detect several protein molecules of interest, including the spike protein associated with COVID‐19. Streptavidin (StP), a protein with a molecular weight of 66 kDa, was employed as a standard biomolecule to be detected on the surface of the b‐As/SnS_2_ NDR device using a biotin‐conjugated pyrene moiety as a support assembly, as shown in **Figure** **5**a. The biotin‐conjugated pyrene moiety synthesis mechanism is described in detail in Figure S3 (Supporting Information). Moreover, the successful coupling of the pyrene moiety was confirmed via middle‐ and long‐wave UV spectroscopy (see Figures S4a,b, Supporting Information). A schematic of the NDR device used as a biosensor is shown in Figure 5b. Pyrene rings loaded with lysine and biotin (PLB) were used as supporting molecules to prepare NDR devices for the detection of streptavidin protein. For functionalization, a small droplet of dimethylformamide (DMF) solution containing PLB (1 nm) was dropped over the channel of the NDR device containing the b‐As/SnS_2_ vdW heterostructure. The surface of the NDR device provides an appropriate stacking opportunity for the hexagonal pyrene ring because of the *π*–*π* bonding with the top SnS_2_ sheet without lying over its surface, as shown in Figure 5b. This device functionalization scheme provides self‐oriented and directionally controlled *π*–*π* stacking of the PLB molecules while avoiding non‐specific interactions. Functionalization with the PLB molecule and capture of streptavidin by b‐As and SnS_2_ sheets were pre‐examined by Raman spectroscopy, as shown in Figure S5 (Supporting Information).

As illustrated in Figure 5c, the NDR device was first tested at a bias voltage of *V*_ds_ = 0.5 and 1 V at a fixed *V*_g_ = 40 V to conduct real‐time streptavidin detection. The NDR device exhibited a high current response at *V*_ds_ = 0.5 V, as indicated by the NDR peak which was observed at this bias voltage. For the other streptavidin‐detection measurements, the NDR device was set to a fixed bias voltage *V*_ds_ = 0.5 V and gate voltage *V*_g_ = 40 V. For the successful capture of the target protein, the device was functionalized using the PLB molecules followed by equilibration. It can be seen that during functionalization, as the PLB molecules are attached to the surface of the NRD device, a sharp response current is recorded as a consequence of a huge amount of charge transfer from the PLB molecules to the b‐As/SnS_2_ vdW heterostructure.^[^ ^16^, ^42^, ^43^ ^]^ The response current was measured until it was saturated. Furthermore, to eliminate the unattached and excessive PLB supporter molecules and to set the baseline for protein detection, the device was washed with deionized water (DIW). As shown in Figure 5d, the current levels before and after washing were approximately the same, owing to the pre‐optimized concentration of PLB (1 nm). This reflects a well‐saturated and homogeneous functionalization. For the real‐time detection of streptavidin, the baseline current level was considered as a reference.

To detect the target protein at various concentrations, a solution containing 1 pm streptavidin was poured over the NDR device using a micropipette, and the normalized current response (*I*_res_ = (*I–I*_o_)/*I*_o_ = Δ*I*/*I*_o_) was recorded as a function of time. Streptavidin was successfully captured by the PLB molecules because of its strong non‐covalent interaction with biotin, resulting in a gradual decline in the device current, as shown in Figure 5d. This decrease in current is attributed to the bonding of streptavidin with the PLB supporter molecules. When PLB molecules capture streptavidin molecules, the charge of the PLB molecules is shared with the streptavidin molecules because of the strong non‐covalent bonding of streptavidin with biotin, which is coupled to the support assembly of the pyrene‐lysine. The binding of streptavidin to biotin is due to their inherent strong bonding ability, with a dissociation constant of the order of 10^−14^ mol L^−1^.^[^ ^38^, ^44^ ^]^ The concentration of streptavidin was varied from 1 pm (in step *I*) to 100 pm in sequence by removing the previous solution and drop‐casting the fresh solution containing 10, 30, and 50 pm streptavidin in steps *III, IV*, and *V* followed by 9 pm in step *II*. The corresponding normalized currents for each concentration were recorded, as presented in Figure 5d. In comparison with all known competitive detection techniques, we accomplished highly sensitive and specific target (streptavidin) detection down to 1 pm concentration, ensuring a precise and rapid response.^[^ ^38^ ^]^ **Table** **1** shows a detailed comparison of the NDR device performance with that of various other biosensors.

**Table 1:** Comparison of NDR biosensor device performance with various types of biosensors for biomolecule detection

Sensor type	Materials	Target	Detected concentration	Response time	References
Electro‐chemical	PdNPs/Si ITO	Dopamine	25 nm	–	[45]
Metal‐mesh	Ni and Si	Streptavidin	900 nm	2 min	[44]
FET	WSe_2_	Streptavidin	1 pm	2 min	[46]
FET	rGO	DNA‐influenza	5 pm	2–4 h	[47]
FET	MoS2	Streptavidin	1 fm	23 min	[48]
vdW BJT	MoTe_2_/GeSe/MoTe_2_	Streptavidin	5 pm	7.5 s	[49]
FET	Graphene	Exosomes	0.1 µg mL^−1^	30 min	[42]
Anodic alumina	ATPES	Streptavidin	1 µg mL^−1^	10 min	[50]
FET	Si‐nano wires	Streptavidin	2 pm	80 min	[38]
NDR device	b‐As/SnS_2_	Streptavidin	1 pm	12.5 s	This work*

* is indicating the significance of results.

The prompt real‐time response of the NDR device was analyzed during the detection of streptavidin, as shown in Figure 5e. All of the NDR devices demonstrated almost 90% of the ultimate time response within 12.5 s. We also examined the selectivity of the NDR device for a specific protein. The device response percentage was defined as (Δ*I*/*I*_o_) × 100, which represents the relative decrease in the response current of the NDR device. The response percentage was estimated for various concentrations of streptavidin, ranging from 1 to 100 pm, as shown in Figure 5f. The response percentages to the supporter molecule (in black), non‐targeted protein (in gray), and streptavidin (in blue) are plotted as a function of the picomolar concentrations, with error bars of 95% accuracy. The protein selectivity of the NDR device was confirmed using bovine serum albumin (BSA, non‐targeted protein), which has the same molecular weight as that of streptavidin (target protein). For BSA concentrations similar to those of streptavidin, the NDR device did not exhibit a significant response percentage. In contrast, a gradual increase in the response percentage was observed as the concentration of streptavidin was increased from 1 to 100 pm. This increase in response percentage indicates that more streptavidin molecules interact with the PLB supporter molecules because of the strong bonding between streptavidin and biotin present in the PLB, thereby reducing the charge carrier density of the NDR device.

## 3 Conclusion

We fabricated an atomically thin vdW stacking structure composed of p‐type b‐As and n‐type SnS_2_ to build a type‐III (broken gap) heterojunction, leading to a negative differential resistance (NDR) device. The vdW heterostructure exhibited nice Esaki‐diode NDR characteristics even at room temperature with a peak‐to‐valley current ratio (PVCR) of 2.8 at *V*_g_ = +40 V. Charge transport through the NDR device was investigated under electrostatic gating to achieve a high PVCR value, which improved from 2.8 to 4.6 when the temperature was lowered from 300 to 100 K. At various applied‐bias voltages, all conceivable tunneling mechanisms that regulate the charge transport were extensively elucidated. Charge transport was attributed to BTBT, TE, and FN tunneling as the bias voltage was swept from the negative to positive regime. Furthermore, the NDR device was deployed as a biosensor for the first time to detect a selective protein as an analyte within 12.5 s, anticipating its potential applications. The real‐time response of the NDR device was investigated at a lower power using various concentrations of the target protein ranging from 1 to 100 pm. Such potential applications of the NDR device may lead to cutting‐edge electronic devices in the near future that can be used as biosensors to detect a wide range of target DNA and protein molecules, including the spike protein associated with COVID‐19.

## 4 Experimental Section

### Fabrication of the NDR device

High‐quality grains of p‐type b‐As and n‐type SnS_2_ materials were obtained from a 2D semiconductor manufacturer (3260 N. Hayden Rd Suite 210‐380 Scottsdale, AZ 85251, USA) to fabricate the NDR device. Small chunks of both 2D semiconductor materials were exfoliated into few‐layer thick sheets using a residue‐free piece of transparent scotch tape and transferred over transparent polydimethylsiloxane (PDMS) stamps. For the formation of clean and sharp vdW heterostructures, uniformly thin layers of both materials were selected and transferred onto ultra‐clean p‐doped substrates of Si/SiO_2_. A dry transfer technique was adopted to transfer the few‐layer‐thick SnS_2_ sheet over the b‐As sheets by means of vdW stacking. Subsequently, photolithography and electron beam lithography systems were utilized to design the metal electrode over the SnS_2_ and b‐As sheets. Finally, Cr/Au (3/50 nm) metal electrodes were deposited in a high‐vacuum metal deposition chamber, followed by lift‐off.

### Characterization of the van der Waals Heterostructure

To analyze the quality and surfaces of the SnS_2_ and b‐As sheets, a Raman Renishaw machine with a laser beam with a wavelength of 532 nm was utilized. The Raman analysis results for both materials are presented in Figure 1f,g and Figures S5 and S6 (Supporting Information). Furthermore, the surface roughness and thickness of each sheet were analyzed under a non‐contact mode atomic force microscope, as shown in Figure 1c,e.

### Electrical Characterizations

To investigate the charge transport through the b‐As/SnS_2_ vdW stacking, all devices were electrically characterized in vacuum. Two Keithley K‐2400 multimeters were used for electrostatic gating and bias voltages, and a Keithley K‐6485 picoammeter was employed to measure the electrical currents. For streptavidin detection, all real‐time measurements were performed under ambient conditions. The bias voltage was fixed at 0.5 and 1 V, and the gate voltage was set to +40 V.

### Statistical Analysis

GraphPad Prism 8 and OriginPro 9.0 were used for graphing and visualizing the data. When analyzing the Raman spectra, the peak characteristics (resonance, FWHM and defects) were approximated with OriginPro 9.0 software using a multi‐peak Gaussian fit utilizing, as shown in Figure 1f,g. For all fittings, the coefficient of determination (*R* ^2^) was greater than 98%. To estimate the PVCR under various gate voltages, the values for three different devices were measured for consistency (Figure 2d). The variance (*P*) for all sets of measurements was ≤0.02, indicating highly reliable data. Moreover, the mean of the squared successive differences (MSSD) was <0.008, confirming the significance of the data. The interquartile range (IQR) was <0.25 and the skewness was zero. The details of the statistical analysis can be seen in Table S1 (Supporting Information). Similarly, the PVCR was plotted at various temperatures. At each temperature, the measurement was repeated using three different devices (*n* = 3), as shown in Figure 3c. The standard error (SE) of the mean was <0.004, with a standard deviation (*σ*) of 0.007, IQR of 0.14, and MSSD < 0.00005, while the *p* was ≤0.0005. Overall, for all sets of temperature measurements, the *σ* was <0.007 with IQR ≤ 0.01 and MSSD ≤ 0.00006. The *P* for all sets of measurement was ≤0.00005 (normally a *p* value ≤ 0.05 is considered significant). The details of the statistical analysis can be seen in Table S2 (Supporting Information). Finally, the measurement of the real‐time response of the NDR device (Figure 5f) was repeated with *n* = 3. For all measurements, *p* was significant (≤0.05) with *σ* ≤ 0.2, IQR ≤ 0.4, and MSSD ≤ 0.02. The details of the statistical analysis can be seen in Table S3 (Supporting Information). When compared to non‐targeted proteins of similar concentration, 1 pm of the target protein (streptavidin) was detected with a reliable statistical analysis, considering the mean ± 5 × *σ*. Statistically, for 1 pm BSA the response percentage was −4.98 ± 5 (0.21) = −3.93% < 0 < −6.03%, whereas for baseline (functionalization) the response percentage was 0.502 ± 5 (0.171) = −0.353% < 0 < +1.357%. Minitab‐17 software was used to conduct all the statistical analyses.

## Conflict of Interest

The authors declare no conflict of interest.

## Author Contributions

This project was designed by G.D. and J.E. G.D. fabricated the devices and characterized the materials. Z.M.S. synthesized the support PLB molecules, A.R. assisted for laboratory analysis. G.D., S.N., and Z.M S. extracted the data. G.D., D.K., Z.M.S., M.U., L.W., S.N., S.H.J., and J.E. analyzed the data, and G.D. and J.E. supervised the project and wrote the manuscript. G.D. also shared the only 1st authorship equally with S.N.

## Supporting information

Supporting Information

## Data Availability

The data that support the findings of this study are available from the corresponding author upon reasonable request.
