# Supplementary material for: Low‐Power Negative‐Differential‐Resistance Device for Sensing the Selective Protein via Supporter Molecule Engineering
Source: Adv Sci (Weinh). 2022 Nov 14;10(1):2204779. doi: 10.1002/advs.202204779 (PMC9811440; doi:10.1002/advs.202204779)
Supplement: Supplementary file 1 — Supporting Information [file ADVS-10-2204779-s001.pdf]

ADVANCED SCIENCE
Open Access

Supporting Information

for *Adv. Sci.*, DOI 10.1002/advs.202204779

Low-Power Negative-Differential-Resistance Device for Sensing the Selective Protein via Supporter Molecule Engineering

Ghulam Dastgeer, Sobia Nisar, Zafar Muhammad Shahzad, Aamir Rasheed, Deok-kee Kim, Syed Hassan Abbas Jaffery, Liang Wang, Muhammad Usman and Jonghwa Eom**

Supporting Information

Low-Power Negative-Differential-Resistance Device for Sensing the Selective Protein via Supporter Molecule Engineering

Ghulam Dastgeer[1 †], Sobia Nisar[2 †], Zafar Muhammad Shahzad[3,4], Aamir Rasheed[5], Deok-Kee Kim[2], Syed Hassan Abbas Jaffery[6], Liang Wang[7], Muhammad Usman[7], Jonghwa Eom*[1]*

[1]Department of Physics and Astronomy, Sejong University, Seoul 05006, Korea

[2]Department of Electrical Engineering, Sejong University, Seoul 05006, Korea

[3]Department of Chemical & Polymer Engineering, University of Engineering and Technology, Lahore, Faisalabad Campus, 38000, Pakistan

[4]SKKU Advanced Institute of Nanotechnology (SAINT), Sungkyunkwan University, Suwon 16419, Korea

[5]Department of Physics and Interdisciplinary Course of Physics and Chemistry, Sungkyunkwan University, Suwon, Gyeonggi-do 16419, Republic of Korea

[6]HMC (Hybrid Materials Center), Department of Nanotechnology and Advanced Materials Engineering, and Graphene Research Institute, Sejong University, Seoul 05006, Korea

[7]Department of Bioinformatics, School of Medical Informatics and Engineering, Xuzhou Medical University, Xuzhou, 221006, China

Corresponding authors: *Jonghwa Eom and Ghulam Dastgeer*

Email: eom@sejong.ac.kr and gdastgeer@sejong.ac.kr

Electrical characterization of b-As and SnS_2

The electrical characteristics of each material were analyzed to investigate their intrinsic nature. At a fixed V_{ds} of 0.5 V, the gate-dependent transfer curves indicated the n-type and p-type natures of SnS_2 and b-As, respectively, as shown in **Figures S1a** and **S1b**. The charge carrier densities of the electrons in SnS_2 and holes in b-As were estimated using $n = q^{-1}C_{ilg}|V_{th} - V_{ilg}|$ [1-2]. The electron carrier density was calculated to be approximately $2.1\times10^{11}/cm^2$ at $V_g = 0$ V, with a high on/off current ratio of $\sim10^5$, while the hole carrier density in b-As was estimated to be approximately $1.12\times10^{11}/cm^2$ at $V_g = 0$ V. The output curves for SnS_2 and b-As were investigated, and the non-linear I_{ds}-V_{ds} curves at zero gate voltage show the Schottky barrier contribution of the Cr/Au electrodes for both SnS_2 and b-As (**Figures S1c** and **S1d**).

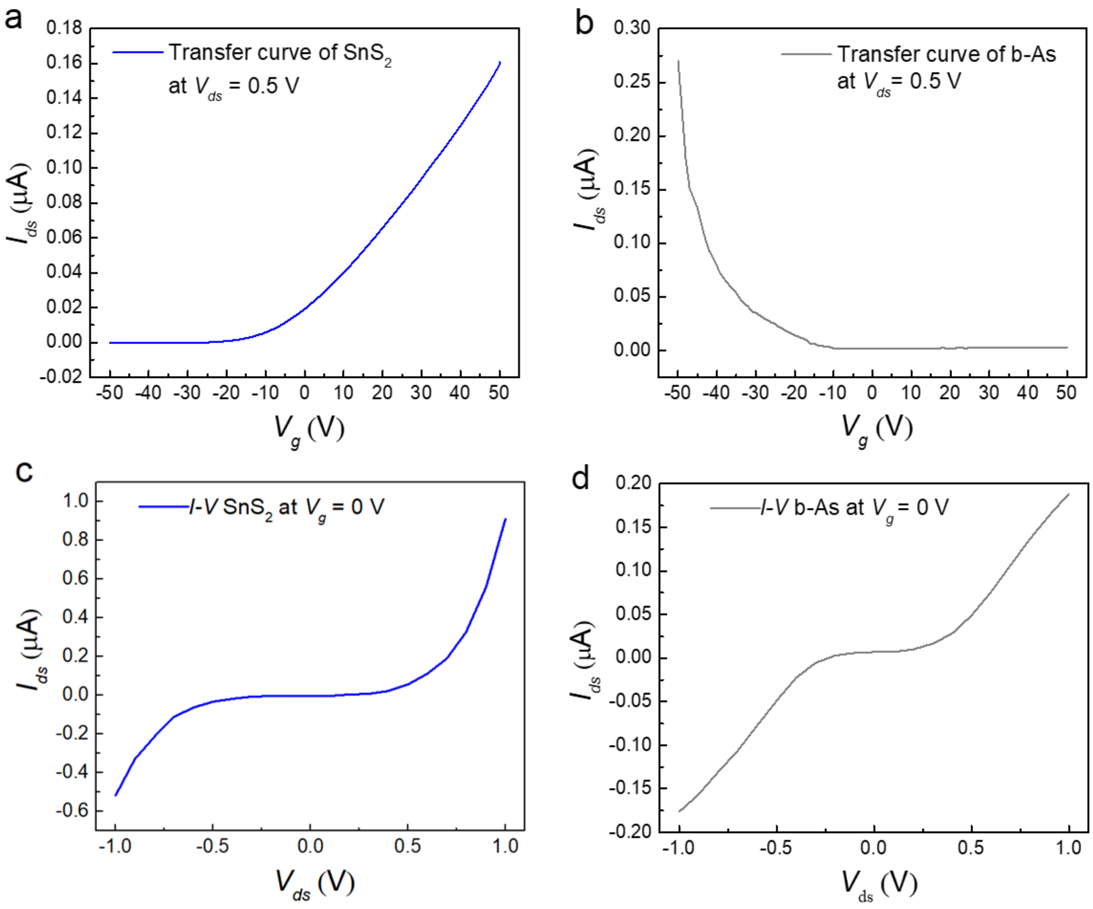

Figure S1. (a) The gate-dependent transfer curve extracted from the n-type SnS_2 flake and **(b)** p-type b-As, at a fixed bias voltage of V_{ds} = 0.5 V. **(c)** Output curve of n-type SnS_2 and **(d)** p-type b-As flake, at zero gate voltage and room temperature.

Furthermore, charge transport through the vdW heterostructure was studied at low temperatures to verify the tunneling mechanism between b-As/SnS_2. If the plot of ln (I/V^2) vs. ($1/V$) shows an exponential increase at higher V_{ds} then it represents direct tunneling, whereas the linear trend of the ln (I/V^2) vs. ($1/V$) plot verifies Fowler–Nordheim (FN) tunneling. **Figures S2a** and **S2b** illustrate the linear trend at higher V_{ds} values, which confirms FN tunneling at a fixed gate voltage of 40 V. The *I-V* characteristics were measured at 100, 200, and 300 K to verify the FN tunneling.

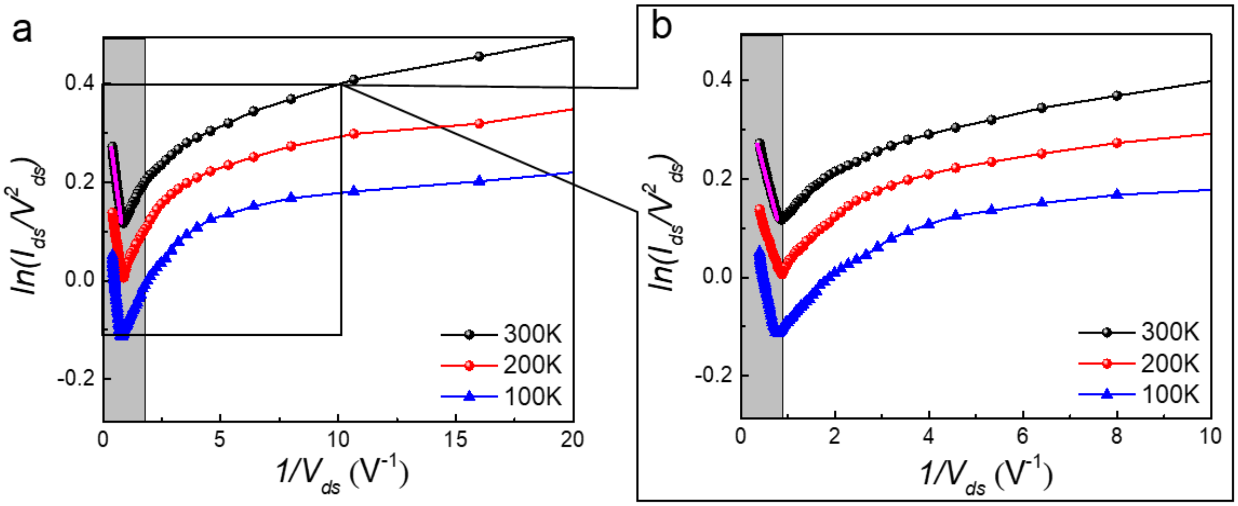

Figure S2. (a) The ln (I/V^2) vs. ($1/V$) plot at various temperatures ranging from 100 K (blue color) to 300 K (black color), at a fixed V_g = 40 V. **(b)** The ln (I/V^2) vs. ($1/V$) plot in the small range of the bias voltage. The slope line (pink color) at the higher bias region is drawn for the curve extracted at 300 K.

PLB synthesis and reaction mechanism

The PLB supporter molecule (pyrene_lysine_biotin construct) was synthesized by a manual solid-phase peptide synthesizer (SPPS), using Fmoc chemistry-based H-Rink amide ChemMatrix resin (0.54 mmol/g substitution value, PCAS BioMatrix Inc.). The 250 mg of resin (estimated based on a 0.14 mmol synthesis scale) was swelled in DMF for 30 min before starting the synthesis. The synthesis was initiated by applying a deprotection reaction. For de-protection, 20% piperidine in N, N-dimethylformamide (DMF) was used, and a standard set of conditions was used, that is, 75 °C ± 5 for 15 s and 90 °C ± 5 for 50 s, respectively. The main washing (repeated three times) was carried out using DMF and alternatively using dichloromethane (DCM) between the deprotecting and coupling steps. Starting the synthesis, 0.2 M Fmoc-Lys (Biotin)-OH solution (prepared using supplier protocol) was coupled with activated amino acid (DIPEA), using DMF as a solvent. The coupling reaction, assisted by 0.5 M diisopropylcarbodiimide (DIC) and 1 M Oxyma, was carried out in a solid-phase peptide synthesis (SPPS) synthesizer at 90 °C ± 5 for 110 s followed by 75 °C ± 5 for 15 s, with alternating steps of washing with DMF and DCM. After de-protection, the same concentration (0.2 M) of 1-pyrene butyric acid was utilized for the coupling reaction under the conditions mentioned above. The final product was then dried in a desiccator for 1.0 h. The product was cleaved from the solid support using a cleavage solution containing trifluoroacetic acid (TFA), tri-isopropyl silane (TIS), and H_2O (95:2.5:2.5). The resulting solution was stirred at room temperature for 2 h. After stirring, the cleavage solute was filtered and separated from the resin under pure nitrogen flow. Subsequently, the product was precipitated in cold diethyl ether and lyophilized using (Labconco). The reaction mechanism is explained in detail in **Figure S3**. Confirmation of coupling and deprotection was performed at each stage of synthesis using the

Kaiser test. Finally, the concentration of the support construct was calculated by measuring absorbance at 335 nm. For substrate functionalization and to avoid stacking, an optimized concentration of the support molecule (1 nM) was used.

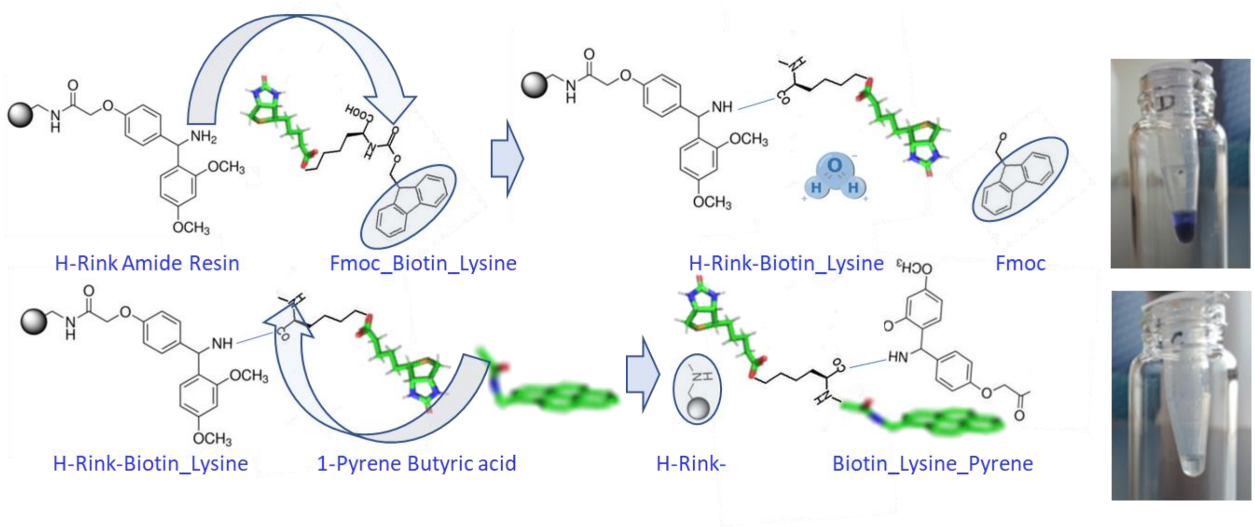

Figure S3. PLB reaction mechanism. Step 1: the de-protected H-Rink amide resin is coupled with Fmoc-protected biotin_lysine (0.2 M solution). Step 2: the coupling reaction of 1-pyrene butyric acid via standard coupling conditions and the final cleavage. For coupling, a standard coupling solution containing DIC and Oxyma was used, while for de-protection, 20% piperidine in DMF was used. In all reactions, the coupling (colorless beads) and de-protection (violet color beads) were confirmed by the Kaiser test.

Material characterization

Solid unconjugated streptavidin was purchased from Thermo Fisher Scientific, and a 100 µM solution was prepared, followed by serial dilutions using deionized water (DIW). The concentration was confirmed by measuring the absorbance of the solution (at 280 nm) using a spectrophotometer (**Figure S4a**) and calculating the concentration using the Beer–Lambert law. The synthesized PLB construct was characterized by UV spectroscopy, as shown in **Figure S4b**.

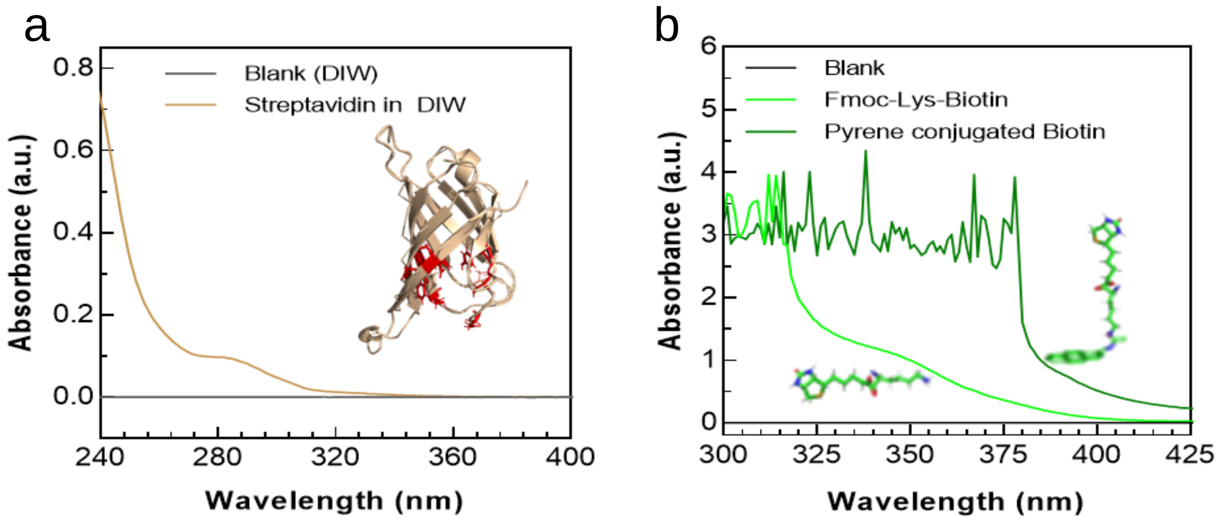

Figure S4. (a) UV-Vis spectrum of streptavidin in the solution phase. The concentration was estimated by measuring the absorbance at 280 nm and using the Beer–Lambert law. **(b)** The UV spectrum of the supporter construct used to detect the conjugation of pyrene to lysine-biotin by SPPS. The sharp peak at ~335 nm shows the presence of pyrene.

Raman spectra analysis of channel material

Raman spectra were recorded at room temperature using a 532 nm laser via a 50x (100x) objective lens. The spectra were recorded for a device containing pristine SnS_2, after functionalization with our pyrene-based receptor, and finally after capturing the target biomolecule (streptavidin). The spectra of bare SnS_2 show clear and sharp peaks at ~315.4 cm^{-1} (A_{1g}) and ~200 cm^{-1} (E_g), indicating the pristine nature of the material[3-4]. These peaks represent two Raman-active phonon modes of 2H-phase SnS_2[5]. The presence of a single peak in the 190–225 cm^{-1} range represents pure single-crystal SnS_2 in its ground state with 2H polytype. By applying a Gaussian fit, the full width at half maximum (FWHM) of the main resonance peak (A_{1g}) was estimated to be 9.85, representing the crystalline nature of the material. Furthermore, the sharp second resonance peak at 200 cm^{-1} represents a small number of layers of SnS_2, whereas the broad peak at this position is attributed to a large number of layers. Because of the pristine nature of the SnS_2 material, the intensity ratio A_{1g}/E_g was very high (101.75), as shown in **Figure S5a**. Moreover, the properties of the peaks were plotted for three different positions and are consistent in **Figure S5b**. However, after functionalizing pure SnS_2 with our pyrene-based supporter construct, the peak properties changed significantly. The crystallinity represented by A_{1g} is absent, which results in an increase in its FWHM from 9.85 to 10.40. Moreover, the second resonance peak (E_g) appears predominantly, representing the presence of a multilayer due to the attachment of our supporter construct onto SnS_2, eventually resulting in a sharp decrease in the peak intensity ratio $A1_g/E_g$ from 101 to 36.80. Furthermore, upon applying the solution containing our target protein, the functionalized channel successfully captured it, owing to the inherent binding affinity between biotin and streptavidin. Because of the streptavidin capture, the FWHM of the A_{1g} peak increased to 12.70, and the peak intensity ratio

(A_{1g}/E_g) further decreased to 19.07. The results were consistent for multiple measurements, as shown in **Figure S5b**.

Similarly, the Raman spectra of the device channel containing mechanically exfoliated b-As were recorded at room temperature using a 532 nm laser via a 50x (100x) objective lens. Pure b-As consists of 12 lattice vibrational modes at the G-point, of which six are Raman active with irreducible representation. However, our results revealed two distinct peaks at 225 (B_{2g}) and 257 (A_{2g}) cm^{-1}, consistent with a previously published report [6]. Here, B_{2g} represents the out-of-plane vibrations of the material and A_{2g} represents the in-plane vibrations. After functionalizing b-As with our PLB construct, the peak properties changed significantly. The main resonance peak (A_{2g}) blue-shifted from 256.57 to 253.51 cm^{-1}, indicating a clear increase in the layer thickness upon functionalization. It can also be observed that the intensity ratio A_{2g}/B_{2g} decreases from 1.68 to 1.66 upon functionalization. The shift in the peak position after the attachment of the pyrene-based support is attributed to the sharing of electrons. Pyrene has a higher electron density than b-As, and charge is transferred to b-As upon functionalization. The weakening of the peak intensity and its shifting can be more clearly observed after streptavidin capture. The intensity of A_{2g}/B_{2g} decreased to 1.54, while a clear blue shift in the A_{2g} peak can be observed in **Figure S5c**. This large change is due to the relatively large size of the streptavidin protein (5.2 nm), as compared to the pyrene-based supporter construct (~1.5 nm). The attachment of streptavidin results in the effect of bulk b-As, resulting in broadening of peaks, and this observation is in agreement with previously published literature [7]. The properties of the peaks were plotted at three different positions, as shown in **Figure S5d**.

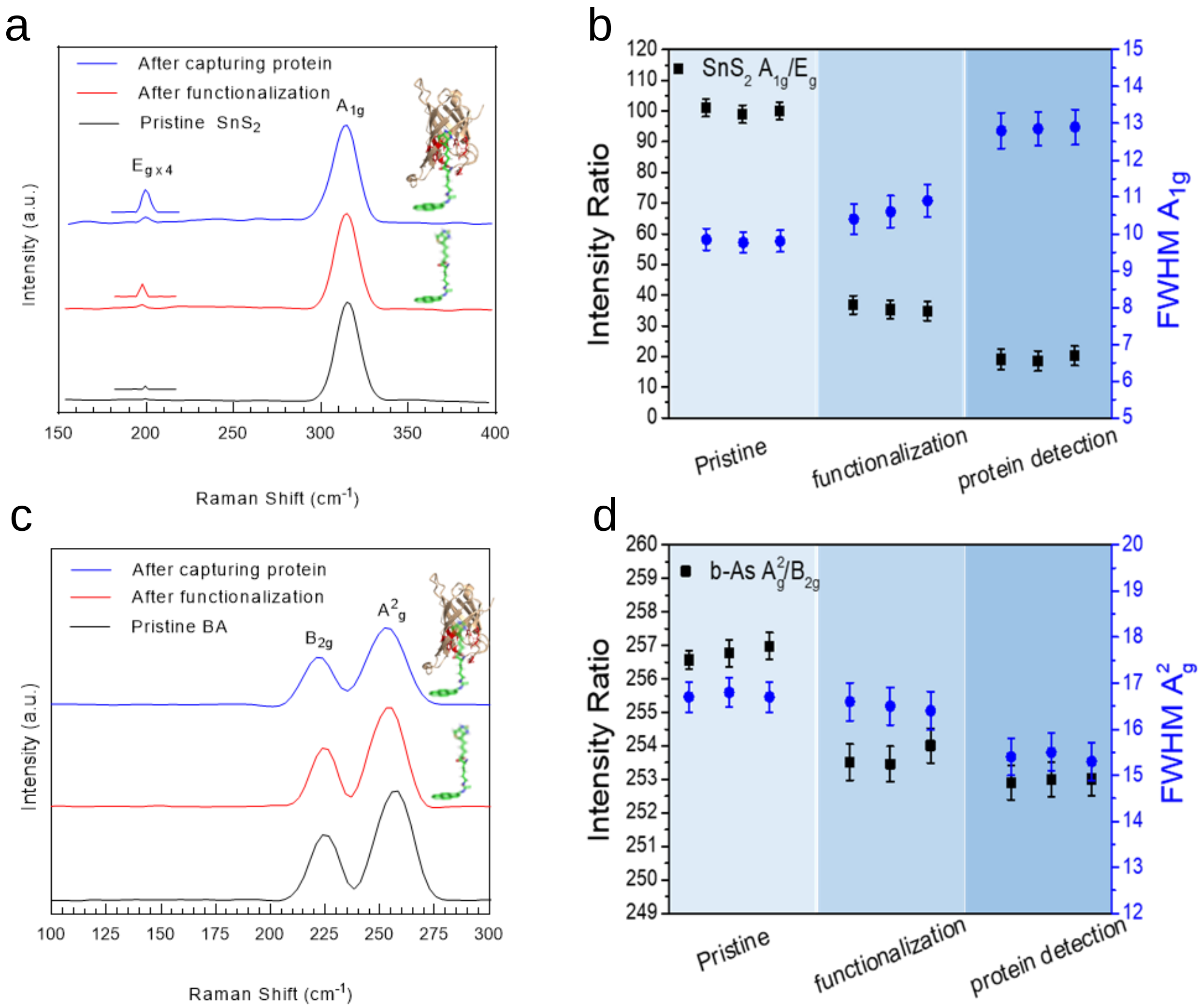

Figure S5. Characterization of SnS_2 and b-As via Raman spectroscopy. **(a)** The Raman spectrum of the pristine SnS_2 sheet is shown by the black line with FWHM of the main resonance peak at 9.85. The red and blue lines represent the Raman spectra of the device after functionalization and after streptavidin detection, respectively. The inset represents the 4x magnified section of spectra containing the Eg peak. **(b)** Peak property analysis at each stage of device operation after repeating measurements. **(c-d)** Raman spectra and peak property analysis for the device containing b-As.

Statistical analysis

Table S1. The details of statistical analysis for **Figure 2d**. Here, the gate voltage was our variable against which the peak-to-valley current ratios (PVCRs) were measured. To avoid errors, each set of measurements was repeated for three different devices.

Descriptive Statistics: -10 V, 10 V, 20 V, 30 V, 40 V

Variable	Mean	SE Mean	StDev	Variance	Sum of Squares	Median	IQR	Skewness	MSSD
-10 V	1.1247	0.0713	0.1235	0.0153	3.8251	1.1250	0.2470	-0.01	0.0076
10 V	1.9410	0.0635	0.1100	0.0121	11.3266	1.9410	0.2200	0.00	0.0061
20 V	2.1840	0.0531	0.0920	0.0085	14.3265	2.1840	0.1840	0.00	0.0042
30 V	2.5960	0.0329	0.0570	0.0032	20.2241	2.5960	0.1140	0.00	0.0016
40 V	2.7640	0.0433	0.0750	0.0056	22.9303	2.7640	0.1500	0.00	0.0028

Table S2. The details of statistical analysis for **Figure 3c**. Here, the temperature was our variable, and its effect on the peak-to-valley current ratio (PVCR) was measured. Each set of measurements was repeated for three different devices to minimize error.

Descriptive Statistics: 100 K, 200 K, 300 K

Variable	Mean	SE Mean	StDev	Variance	Sum of Squares	Median	IQR	Skewness	MSSD
100 K	4.6150	0.00404	0.00700	0.00005	63.8948	4.6150	0.0140	0.00	0.00006
200 K	3.2070	0.00404	0.00700	0.00005	30.8546	3.2070	0.0140	-0.00	0.00006
300 K	2.7327	0.00376	0.00651	0.00004	22.4025	2.7330	0.0130	-0.23	0.00005

Table S3. The details of statistical analysis for **Figure 5f**. Here, the response percentage of our sensor was our variable, which was measured during the device performance testing against various targets (streptavidin) and un-wanted protein (BSA), and each set of measurements was repeated for three different devices.

Descriptive Statistics: Base line (0 pM), Streptavidin (20 pM), BSA (20 pM)

Variable	Mean	SE Mean	StDev	Variance	Sum of Squares	Median	IQR	Skewness
Base line (0 pM)	0.5020	0.0987	0.1710	0.0292	0.8145	0.5020	0.3420	0.00
Streptavidin (20 pM)	54.800	0.122	0.212	0.045	9009.210	54.800	0.424	-0.00
BSA (20 pM)	5.153	0.119	0.206	0.042	79.745	5.153	0.412	0.00

Variable	MSSD
Base line (0 pM)	0.0146
Streptavidin (20 pM)	0.022

References

[1] B. Radisavljevic, A. Kis, *Nature Materials* **2013**, 12, 815.

[2] G. Dastgeer, M. F. Khan, J. Cha, A. M. Afzal, K. H. Min, B. M. Ko, H. Liu, S. Hong, J. Eom, *ACS Applied Materials & Interfaces* **2019**, 11, 10959.

[3] M. S. Zafar, G. Dastgeer, A. Kalam, A. G. Al-Sehemi, M. Imran, Y. H. Kim, H. Chae, *Nanomaterials* **2022**, 12, 1305.

[4] L. A. Burton, T. J. Whittles, D. Hesp, W. M. Linhart, J. M. Skelton, B. Hou, R. F. Webster, G. O'Dowd, C. Reece, D. Cherns, D. J. Fermin, T. D. Veal, V. R. Dhanak, A. Walsh, *Journal of Materials Chemistry A* **2016**, 4, 1312.

[5] T. Pan, D. De, J. Manongdo, A. Guloy, V. Hadjiev, Y. Lin, H. Peng, *Applied Physics Letters* **2013**, 103, 093108.

[6] M. Zhong, Q. Xia, L. Pan, Y. Liu, Y. Chen, H.-X. Deng, J. Li, Z. Wei, *Advanced Functional Materials* **2018**, 28, 1802581.

[7] C. Kamal, M. Ezawa, *Physical Review B* **2015**, 91, 085423.
